# Quantifying Periodontal Inflammatory Burden: A Conceptual Clinical Framework Integrating Periodontal Inflamed Surface Area (PISA) and High-Sensitivity C-reactive Protein (Hs-CRP) for Oral–Systemic Health

**DOI:** 10.7759/cureus.106798

**Published:** 2026-04-10

**Authors:** Andrea Mascolo, Oana Dinculescu, Jessica Bassignani, Soufyane Bensaidi

**Affiliations:** 1 Academics, European Institute for Medical Studies (EIMS), St. Julian's, MLT; 2 EIMS Research Hub, European Institute for Medical Studies (EIMS), St. Julian's, MLT; 3 EIMS Research Hub, European Institute for Medical Studies (EIMS), St. Julian’s, MLT; 4 Department of Periodontology, Centre Hospitalo-Universitaire de Tlemcen, Tlemcen, DZA; 5 Faculty of Medicine, Abu Bekr Belkaid University, Tlemcen, DZA

**Keywords:** high-sensitivity c-reactive protein (hs-crp), oral–systemic health, periodontitis, pisa (periodontal inflamed surface area), systemic inflammation

## Abstract

Periodontitis is a chronic inflammatory disease characterized by biofilm-driven tissue destruction and ulcerated pocket epithelium, which may contribute to systemic low-grade inflammation. While conventional periodontal parameters are essential for diagnosis and staging, they do not directly quantify the biologically active inflammatory surface exposed to the bloodstream. This report proposes a clinically feasible framework integrating the periodontal inflamed surface area (PISA) and high-sensitivity C-reactive protein (hs-CRP) to quantify the oral-systemic inflammatory burden. By synthesizing current evidence, this conceptual clinical framework suggests a standardized observational protocol applicable in routine practice. A three-time-point protocol is proposed: a baseline (T0) assessment of periodontal status, PISA, and hs-CRP; an optional intermediate reassessment (T1) to contextualize inflammatory kinetics; and a follow-up (T2, 4-12 weeks) to evaluate sustained changes after periodontal therapy. The primary outcome focuses on the correlation between changes in PISA and hs-CRP. Ultimately, the integrated PISA-hs-CRP framework provides a biologically meaningful and reproducible method to translate periodontal findings into quantifiable systemic inflammatory metrics, supporting interdisciplinary communication and real-world clinical audits. This report presents a proposed multi-time-point observational clinical audit protocol; no empirical data are reported.

## Introduction

Periodontitis is a chronic inflammatory disease driven by dysbiotic biofilm and host immune response, resulting in progressive destruction of periodontal tissues. A defining biological feature of periodontitis is the presence of ulcerated pocket epithelium, which represents a chronic inflammatory wound capable of facilitating the translocation of bacteria, endotoxins, and inflammatory mediators into the systemic circulation. Consequently, periodontitis is increasingly recognized as a contributor to systemic low-grade inflammation and as a potential modifier of cardiometabolic and other chronic inflammatory conditions [1-3].

Conventional periodontal parameters, including probing pocket depth (PPD), clinical attachment level (CAL), and bleeding on probing (BOP), remain fundamental for diagnosis and staging. However, these measures are largely anatomical or categorical and do not directly quantify the extent of inflamed ulcerated epithelial surface that is biologically relevant for systemic inflammatory exposure [4].

The periodontal inflamed surface area (PISA) was developed to address this limitation by transforming routine periodontal measurements into a continuous metric (expressed in mm²) representing the surface area of bleeding pocket epithelium [4]. PISA therefore provides a quantitative estimate of the active periodontal inflammatory burden.

High-sensitivity C-reactive protein (hs-CRP) is a well-established biomarker of systemic low-grade inflammation and is widely used in medical risk stratification, particularly in cardiovascular medicine [5-7]. Observational studies and meta-analyses indicate that individuals with periodontitis exhibit higher CRP or hs-CRP levels compared with periodontally healthy controls, and that periodontal therapy may reduce systemic inflammatory markers over time [1-3,8-10].

The aim of this article is to propose a pragmatic clinical framework integrating PISA and hs-CRP to quantify oral-systemic inflammatory burden in a standardized and clinically applicable manner.

## Technical report

This is a clinical framework proposal based on a synthesis of current peer-reviewed literature addressing: (i) PISA as a quantitative measure of periodontal inflammatory burden, (ii) associations between periodontitis or PISA and systemic inflammatory biomarkers, including hs-CRP, and (iii) systemic inflammatory responses following periodontal therapy [4-10].

The proposed framework is intended for structured observational clinical audits and real-world practice-based data collection. It is not designed to establish causal inference but rather to generate hypothesis-supporting data within routine periodontal care. The model is deliberately structured to be feasible in everyday clinical settings, without requiring additional invasive procedures beyond standard periodontal examination and venous blood sampling.

Study design

This study is proposed as a prospective, single-arm observational clinical audit with repeated measures conducted in real-world periodontal practice. The framework is designed to assess temporal changes in periodontal inflammatory burden and systemic inflammatory markers following standard non-surgical periodontal therapy.

Objectives

The primary objective is to quantify the correlation between changes in periodontal inflamed surface area (ΔPISA) and changes in high-sensitivity C-reactive protein (Δhs-CRP), with a correlation coefficient of r ≥ 0.3 considered clinically meaningful. Secondary objectives include evaluating changes in bleeding on probing (BOP) and conventional periodontal parameters following therapy.

Proposed clinical framework

Rationale and Clinical Integration

The PISA serves as a robust metric for quantifying the biologically active inflammatory wound surface associated with periodontal disease by integrating PPD, CAL or gingival recession, and BOP into a single continuous variable. This metric reflects current inflammatory activity rather than historical tissue destruction and can be interpreted as a morphometric proxy of the ulcerated inflammatory interface between the periodontal pocket and the systemic circulation.

To ensure accuracy, PISA values should be calculated according to the original method described by Nesse et al. [4]. This requires full-mouth periodontal charting using a standardized periodontal probe applying approximately 25 g of probing force, with PPD and BOP recorded at six sites per tooth (mesiobuccal, mid-buccal, distobuccal, mesiolingual, mid-lingual, distolingual) in accordance with established periodontal examination protocols. Electronic spreadsheets or structured digital tools may be used to facilitate these calculations, provided that they must strictly adhere to the published formula and full-mouth standardized periodontal charting protocols.

Complementing this local metric, hs-CRP provides a systemic measure of low-grade inflammation. It is widely used in medical practice with established interpretive thresholds: low risk (<1 mg/L), intermediate risk (1-3 mg/L), and high risk (>3 mg/L) [5-7]. Integrating PISA and hs-CRP allows clinicians to evaluate whether reductions in the local periodontal inflammatory burden are accompanied by measurable changes in systemic inflammation. 

Setting and Participants

The framework is intended for implementation in periodontal clinical settings, including university-based and private practice environments, with the following eligibility criteria: Inclusion criteria: (i) Adults ≥18 years with stage II-IV periodontitis according to the current periodontal classification and (ii) with no acute systemic infection. Exclusion criteria: (i) Recent antibiotic or anti-inflammatory therapy and (ii) Pregnancy or systemic inflammatory diseases affecting CRP levels

Recruitment

Participants to be consecutively recruited from patients attending routine periodontal care and may be stratified post hoc according to systemic comorbidities (e.g., diabetes, smoking status).

Measurement and Unit of Analysis

The unit of observation and analysis would be the patient-level PISA, derived from full-mouth six-site periodontal charting. Standardized periodontal probing will be performed using calibrated probes with a force of 20-25 g. Examiner calibration will be conducted to ensure inter-examiner reliability (target ICC >0.85). hs-CRP will be measured using standardized laboratory assays under fasting conditions.

Therapy Description

To maintain consistency within this framework, periodontal therapy is defined as non-surgical full-mouth instrumentation (scaling and root planing or equivalent mechanical debridement) performed within a one to two-week window, avoiding adjunctive systemic anti-inflammatory medication unless clinically necessary. The structured implementation of these assessments across different stages is detailed in the Time-Point Protocol (Table 1).

**Table 1:** Time-point protocol within the proposed PISA–hs-CRP framework PPD: probing pocket depth; CAL: clinical attachment level; BOP: bleeding on probing; PISA: periodontal inflamed surface area; hs-CRP: high-sensitivity C-reactive protein

Time Point	Clinical Procedures
T0 (Baseline)	Full-mouth periodontal charting (PPD, CAL/recession, BOP) using standardized six-site-per-tooth assessment with calibrated probe (20–25 g force). Calculation of PISA according to Nesse et al. [4]. Standardized venous blood sampling for hs-CRP (morning collection, fasting when feasible, absence of acute infection).
Treatment Phase	Non-surgical full-mouth periodontal instrumentation (scaling and root planing or equivalent mechanical debridement) performed within a defined treatment window (approximately 1–2 weeks).
T1 (Optional Early Reassessment, 7–14 days)	Optional reassessment for contextualizing early inflammatory kinetics. Measurements obtained within 24–48 hours after intensive instrumentation should be interpreted cautiously due to possible transient systemic inflammatory responses [3,8].
T2 (Follow-up, 4–12 weeks after therapy)	Repeat full-mouth periodontal assessment sufficient to recalculate PISA. Repeat standardized hs-CRP measurement. The 4–12 week interval is consistent with commonly reported post-therapy evaluation periods in non-surgical periodontal studies.

Standardized protocol and data interpretation

The reliability of this framework depends on the standardization of biological sampling and the timing of assessments. Given the known intra-individual variability and potential skewed distribution of hs-CRP values, blood sampling should ideally occur during morning collection in a fasting state and in the absence of acute infectious conditions. While research settings may require repeated baseline measurements for maximum robustness, a single standardized measurement is generally considered acceptable for routine clinical audits when interpreted with appropriate caution.

Furthermore, the evaluation of success relies on clearly defined endpoints and patient stratification. The primary endpoint of this framework is the correlation between the change in PISA (ΔPISA) and the change in hs-CRP (Δhs−CRP) from baseline (T0) to follow-up (T2). Secondary endpoints include changes in BOP, conventional periodontal parameters, and patient-reported outcomes.

For enhanced interpretative clarity, patients should be stratified into two profiles: those with a predominantly local inflammatory burden and those with a systemic comorbidity profile (e.g., diabetes, obesity, or smoking) known to influence hs-CRP levels. This stratification does not eliminate confounding variables but significantly improves the transparency and clinical interpretability of the results during real-world clinical audits.

Statistical analysis

The primary statistical analysis should assess the correlation between ΔPISA and Δhs-CRP, using either Pearson or Spearman correlation coefficients, depending on the distribution of the data. In the context of repeated-measures implementations, mixed-effects models may be considered to account for intra-subject correlation over time. Hs-CRP values may be log-transformed if not normally distributed. Additionally, multivariable regression models may be used to adjust for potential confounding factors, including age, smoking status, and relevant systemic conditions. Missing data should be reported transparently and managed using appropriate predefined statistical methods.

## Discussion

PISA provides a biologically grounded estimate of periodontal inflammatory burden by quantifying the ulcerated epithelial surface area actively involved in inflammation [4]. Unlike traditional periodontal indices, which may reflect cumulative or historical tissue damage, PISA focuses on current inflammatory activity and therefore has direct relevance for systemic exposure.

Multiple studies have demonstrated associations between PISA and systemic inflammatory markers, including hs-CRP, supporting the concept that periodontal inflammation contributes to overall systemic inflammatory load [1,2,11,12]. Meta-analyses further indicate that periodontal therapy can reduce systemic CRP levels, although short-term increases may occur immediately after instrumentation due to transient bacteremia and inflammatory response [3,8-10].

Beyond cardiometabolic implications, experimental and translational evidence suggests that chronic oral inflammation may contribute to systemic immune activation affecting distant organs. Pro-inflammatory mediators released during persistent periodontal infection can reach systemic circulation and modulate endothelial and immune responses at extra-oral sites, supporting the concept of periodontitis as a contributor to multi-organ inflammatory burden rather than a purely localized disease [13]. However, such mechanisms remain largely hypothetical and exceed the scope of routine clinical biomarkers, reinforcing the need for pragmatic, quantifiable measures such as PISA and hs-CRP in real-world periodontal practice.

The integration of PISA and hs-CRP may therefore enhance interdisciplinary communication by providing a shared quantitative framework linking oral and systemic inflammation. For patients, translating periodontal findings into measurable systemic relevance may improve understanding and adherence. 

From a methodological perspective, the proposed framework could also be positioned within the emerging concept of reverse evidence-based dentistry (rEBD) [14], where structured real-world clinical data contribute to evidence generation. By standardizing the assessment of periodontal inflammatory burden, the PISA-hs-CRP model may help generate practice-based evidence to inform future prospective and interventional studies. Rather than replacing controlled trials, such approaches complement traditional hierarchies of evidence by strengthening transparency, reproducibility, and clinical interpretability in routine practice settings.

Limitations

This framework does not establish causality and is subject to confounding factors such as smoking, obesity, diabetes, medications, and intercurrent inflammatory conditions that independently influence hs-CRP levels [1-3]. Variability in periodontal treatment protocols may also affect short-term inflammatory responses. Prospective studies using standardized PISA-hs-CRP methodologies are needed to further validate clinical thresholds and longitudinal behavior.

## Conclusions

The integrated use of PISA and hs-CRP offers a clinically feasible and biologically meaningful method to quantify oral-systemic inflammatory burden. This framework supports standardized clinical audits, facilitates interdisciplinary collaboration, and contributes to real-world evidence generation on the systemic relevance of periodontal disease, while avoiding over-interpretation beyond available clinical biomarkers.
